# Role of Different *Pf*crt and *Pf*mdr-1 Mutations in Conferring Resistance to Antimalaria Drugs in *Plasmodium falciparum*

**DOI:** 10.1155/2014/950424

**Published:** 2014-11-11

**Authors:** Zaid O. Ibraheem, R. Abd Majid, S. Mohd. Noor, H. Mohd. Sedik, R. Basir

**Affiliations:** ^1^Pharmacology Unit, Department of Human Anatomy, Faculty of Medicine and Health Sciences, Universiti Putra Malaysia, 43400 Serdang, Selangor, Malaysia; ^2^Department of Medical Microbiology and Parasitology, Faculty of Medicine and Health Sciences, Universiti Putra Malaysia, 43400 Serdang, Selangor, Malaysia; ^3^Department of Hematology, Faculty of Medicine and Health Sciences, Universiti Putra Malaysia, 43400 Serdang, Selangor, Malaysia; ^4^School of Bioscience and Biotechnology, Faculty of Science and Technology, Universiti Kebangsaan Malaysia (UKM), 43600 Bangi, Selangor, Malaysia

## Abstract

Emergence of drugs resistant strains of *Plasmodium falciparum* has augmented the scourge of malaria in endemic areas. Antimalaria drugs act on different intracellular targets. The majority of them interfere with digestive vacuoles (DVs) while others affect other organelles, namely, apicoplast and mitochondria. Prevention of drug accumulation or access into the target site is one of the mechanisms that plasmodium adopts to develop resistance. Plasmodia are endowed with series of transporters that shuffle drugs away from the target site, namely, *pf*mdr (*Plasmodium falciparum* multidrug resistance transporter) and *pf*crt (*Plasmodium falciparum* chloroquine resistance transporter) which exist in DV membrane and are considered as putative markers of CQ resistance. They are homologues to human P-glycoproteins (P-gh or multidrug resistance system) and members of drug metabolite transporter (DMT) family, respectively. The former mediates drifting of xenobiotics towards the DV while the latter chucks them outside. Resistance to drugs whose target site of action is intravacuolar develops when the transporters expel them outside the DVs and vice versa for those whose target is extravacuolar. In this review, we are going to summarize the possible *pf*crt and *pf*mdr mutation and their role in changing plasmodium sensitivity to different anti-Plasmodium drugs.

## 1. Resistance to Antimalaria Drugs 

Emergence of resistant strains of* Plasmodium falciparum *to the well-known conventional antimalarials has worsened the calamity of malaria scourge. Resistance to chloroquine (CQ) is the most catastrophic factor as it is still the cheapest and safest among all other antimalaria drugs [1, 2]. It was originated in 6 different foci in the world distributed among Africa, South-east Asia, and Latin America [3].

Development of drugs resistance relies entirely on the array of biological and atmospheric factors and the drug pressure that each strain had experienced during its evolution [4]. For instance, it took decades to develop resistant strains to CQ while resistance to the electron transport inhibitor, atovaquone, may emerge in tandem with its clinical use [5, 6]. The former requires several mutations in the transporter protein [7] whilst only one point mutation is sufficient to confer the latter [8, 9].

Fixed dose combination strategy was adopted by WHO to overwhelm spread of resistance [1]. For instance, prevalence of lumefantrine (LM) resistance is very low due to its use as a part of fixed dose combination therapy along with artemisinin (ART) [10, 11].

## 2. Intracellular Distribution of Anti-Plasmodium Drugs and Role of Transporter Proteins

Access of antimalaria drugs into their target is a prerequisite for their action. They have different intracellular targets, such as digestive vacuole (DV), cytosol, mitochondria, apicoplast, and parasite membrane [12, 13]. Their intracellular distribution relies on their solubility, potential to permeate cell membranes, and binding affinity to transporters that regulate drugs trafficking through intracellular compartments [14, 15].

Normally, eukaryotic cells evade xenobiotics toxicity through trafficking them into the DVs or lysosomes for further procession or expel them extracellularly [15]. In plasmodium, two types of transporters mediate xenobiotics trafficking to the DV [16]: P-glycoprotein related transporters (which include* pf*mdr-1 (*Plasmodium falciparum *multidrug resistance-1),* pf*mdr-2 and* pf*mrp (*Plasmodium falciparum *multidrug resistance associated protein)) and drug metabolite transporter (DMT) system that is represented by* pf*crt (*Plasmodium falciparum* chloroquine resistance transporter). Drugs are trafficked from the cytosol to the intravacuolar compartment by the former and in the opposite direction by the latter [7, 16, 17]. Their function is inconsistent in all the strains as it depends on the drug selection pressure and type of the mutation possessed by the transporter [8, 18, 19].

Different mutations were observed in different strains of* Plasmodium falciparum *[18]. The mutant transporters increase efficacy of drugs as long as they are shuffled into their target site. In other words, if the drug target is intracytosolic, its potency increases when the transporters prevent its accumulation up in the intravacuolar compartment [20–22].

In this review, we are going to summarize the potential of different* pf*crt and P-glycoprotein transporters on the intracellular distribution of the antimalaria drugs.

## 3. Digestive Vacuole Membrane Transporters: (i) P-Glycoprotein Transporters and (ii) Drug Metabolite System Transporters

### 3.1. P-Glycoprotein Transporters

**Figure 1:**
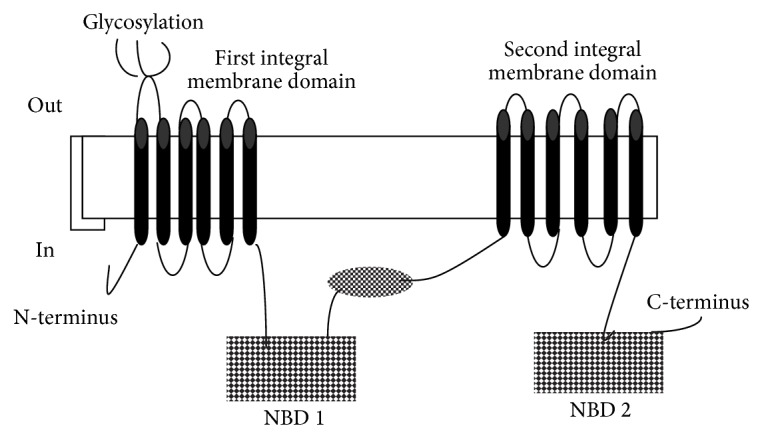
Detailed structure of P-glycoprotein molecule. It is made up of two domains: membrane domain (MD) that is embedded in the DV membrane and nucleotide binding domain (NBD) which faces the cytoplasm and mediates interaction with ATP. When ATP binds to NBD, conformational changes incur in the structure of the molecule resulting in rearrangement of the active sites of the MD domain in such a way that allows accommodation of the substrate molecules and their consequent engulfment throughout the DV membrane.

ABC transporters (ATP dependent cassette transporters) or P-glycoprotein (P-gh) is a group of energy mediated carriers which pump xenobiotics outside the cytosolic compartment [7, 23]. They have active sites that accommodate substrates of dissimilar structure and molecular size (300–2000 KDa) [24]. The majority of the substrates are amphipathic and have at least one aromatic ring attached to an amine group. Their amphipathicity enhances its binding affinity to P-gh as their hydrophobic part binds to the active sites that are embedded inside the membrane while the hydrophilic one binds to the sites exposed to the cytosolic compartment [25] (Figure 1).

Four ABC transporter proteins have been identified in* Plasmodium falciparum,* namely,* pf*mdr 1 (*Plasmodium falciparum *multidrug resistance-1) [26, 27],* pf*mdr 2 (*Plasmodium falciparum *multidrug resistance-2) [28],* pf*mrp (*Plasmodium falciparum *multidrug resistance associated protein) [29],* pf*gcn20, and* pf*a0590w.

*Pf*mdr-1 is ubiquitous on DV membrane and is involved in multidrug resistance through dispatching xenobiotics away of the cytosol [19]. In plasmodium, it acts as an auxiliary mechanism beside simple diffusion for drug entry into the DV.

Meanwhile,* pf*mdr-2 is involved in translocation of heavy metals and has nothing to do with multidrug resistance [30, 31]. Nevertheless, some previous articles had related it to CQR and MQR (mefloquine resistance) [28].

On the other hand,* pf*mrp is present in cell membrane and membrane bound vesicles and is involved in the transport of glutathione and its conjugates. Meanwhile, biological functions of the last two have not been specified yet [29, 32].

#### 3.1.1. Point Mutations in* Pf*mdr-1

*Pf*mdr-1 point mutations were observed in both CQ resistant and susceptible strains of* Plasmodium falciparum. *They ablate the transporter capacity to drift drugs, namely, CQ, quinoline (QN), mefloquine (MQ), halofantrine (HF), and lumefantrine (LM), into the DV [33, 34]. The wild (nonmutant) form (*pf*mdr-1^CQS^) mediates transfer of drugs, such as CQ, quinoline (QN), mefloquine (MQ), halofantrine (HF), and lumefantrine (LM), from the cytosol into the vacuole. Its ubiquity is prominent in MQ, HF, and LM resistant strains as it sways them away from their target site of action in the cytosol [21, 26].

Four plausible single nucleotide polymorphisms (SNPs) were detected in* pf*mdr-1 gene, N86Y, N1042D, S1034C, and D1246Y [33, 35] in which asparagine at codons 86 and 1042, serine at codon 1034, and aspartic acid at codon 1246 of* pf*mdr-1 protein had been replaced by tyrosine, aspartic acid, cystine, and tyrosine, respectively. These substitutions have altered P-gh physiochemical properties as all the substitute amino acids are more polar as compared to their substituent (as indicated by their hydrophobicity indices—Table 1) [36].

Each amino acid has certain physiochemical property, namely, side chain volume, side chain charge, and hydrolipophilicity index. Its substitution in the structure of any transporter changes the transporter physiochemical properties and consequently affects its potential to bind to and transfer different xenobiotic [37].

Distribution of* pf*mdr-1 mutations is inconsistent over different geographic areas due to the discrepancy in the type of stress that each strain had experienced [38]. For instance, N86Y mutation disseminates widely in Asia and Africa while S1034C, N1042D, and D1246Y exist in South America.

#### 3.1.2. Role of the Mutant* Pf*mdr Allele in Drug Resistance

Conflicting results were generated by studies that investigated the correlation between expression level of* pf*mdr-1^CQR^ and the extent of resistance to CQ or other quinoline antimalaria drugs. Earlier studies had revealed that genetic overexpression and protein amplification of the mutant forms of both* pf*mdr-1 and* pf*mdr-2 were significantly correlated to the degree of CQ resistance. Accordingly, scientists had concluded that both* pf*mdr-1 and* pf*mdr-2 induced multidrug resistance which is responsible for CQR in* Plasmodium falciparum* [28]. This conclusion was prejudiced when latter screening studies failed to prove that correlation [39] wherein less copies of* pf*mdr-1^CQR^ were detected in some CQ resistant strains [40, 41]. This led to a suggestion that* pf*mdr-1^CQR^ has a role in augmenting CQR but it is not the sole cause. This observation was proved by one cross-genetic study between HB3, a CQ susceptible strain of* Plasmodium falciparum,* and Dd2, a CQ resistant one. The study showed that transfection of* pf*mdr-1^CQR^ into the susceptible strain or* pf*mdr-1^CQS^ into the resistant strain does not produce a pronounced change in their response to CQ [39].

Furthermore, the majority of the survey studies revealed that there was no correlation between* pf*mdr-1^CQR^ ubiquity and CQR prevalence [34, 38, 41–44].

One epidemiological study that was run in Thailand had found that N86Y is not implicated in CQR as the team failed to find any correlation between its expression and spread of CQR within the screened areas [45]. On the other hand, allelic exchange experiments showed that replacement of these point mutations by wild genes increases susceptibility of the parasite to chloroquine as detected by [3] hypoxanthine incorporation experiments [46].

Eventually, it was concluded that presence of* pf*mdr-1^CQR^ is not provisional for CQR but may relate to fitness adaptations in response to the physiological changes that result from other genetic mutations that are most likely to be associated with* pf*crt.* Pf*crt is another transporting system which was discovered subsequently and is involved in conferring drug resistance. This phenomenon was proved by Reed et al. as the team found that introduction of S1034C, N1042D, and D1246Y mutations through allelic exchange experiments into CQ susceptible strains of* Plasmodium falciparum* did not alter their response to CQ [20, 34, 46]. A similar cross-genetic study run by [47, 48] in which* pf*mdr-1 alleles from each of 7G8, a CQ resistant strain present in Latin America (*pf*mdr-1^7G8^), and D10 strain, a CQ sensitive strain of* Plasmodium falciparum* (*pf*mdr-1^D10^), were transfected to the other strains reciprocally. The results showed that, in spite of the its ability to alter CQ IC_50_,* pf*mdr-1^7G8^ was insufficient to confer CQR in D10 while the wild* pf*mdr-1^D10^ allele could have halved CQR level in 7G8 without changing the parasite phenotype into CQ susceptible.

According to a study by Michael B. Reed, 1999 [48], altering* pf*mdr-1 sequence in 7G8, which is sensitive to MQ, HF, to encode ser 1034, asn 1042, and asp 1246 through transfecting it with a wild form of pfmdr-1 allele (*pf*mdr^D10^), confers for higher resistance to these antimalarials. The most pronounced effect was seen after introducing a single tyr 1246 mutation as compared to that after introducing both asp 1042 and cyc 1034 SNPs. This highlights the importance of 1246 amino acid in the interaction with MQ and LM [47].

Emergence of K1HF strain after exposing K1H, a CQ resistant and HF susceptible cell line of* Plasmodium falciparum,* to HF pressure in study was another proof that CQR is not totally dependent on* pf*mdr-1. K1HF phenotype is alien to that of K1H such that it is CQ susceptible and HF sensitive. This change was not accompanied by any overexpression or sequence change in* pf*mdr [49].

It was found that amplification of the copy number of the wild form of* pf*mdr-1 gene accounts for half of the recrudescence and treatment failure that occur after having selection pressure induced by mefloquine (MQ), lumefantrine (LM), and artemisinin (ART). This phenomenon is accompanied by enhancement in CQ susceptibility [50].

It is noteworthy that there is an interstrain difference in the copy number of* pf*mdr-1 gene that it reaches its upmost values in both Dd2 and FCB strains of* Plasmodium falciparum*. This has an impact on sensitivity to some drugs, such as mefloquine and artemisinin [34].

Overall,* Plasmodium falciparum* strains that exhibit resistance to ART, HF, and/or MQ are characterized by having higher copy number of* pf*mdr-1 allele and ubiquity of the well-known* pf*mdr-1 mutations, namely, 86N, 1034S, 1042N, and 1246N.* Pf*mdr-1 mutations selection may occur after exposure to MQ, ART, and/or HF.

#### 3.1.3. *Pf*mrp-1 (*Plasmodium falciparum *Multidrug Resistance Associated Protein)

*Pf*mdr-1 is not the only ABC transporter protein associated with CQR, but* pf*mrp-1* (Plasmodium falciparum* multidrug resistance associated protein) is another DV membrane protein concerned with CQ inflow into the DV [29]. Genetic survey studies revealed that* pf*mrp ability to drift CQ into the DV is afflicted by two point mutations, Y191H and A437S where tyrosine and alanine at codons 191 and 437 had been replaced by histidine and serine, respectively. The substitute amino acids are polar as compared to their substituent ones as indicated by their values of hydrophobicity index (Table 2). This posits the importance of polarity in the active sites of* pf*mrp for CQ binding. Due to its alkaline characteristics, histidine evolves a steric repulsion with quinine nucleus containing molecules. Although their ubiquity is not provisional for conferring CQ resistance, both of Y191H and A437S mutations play a role in augmenting the degree of CQ resistance [7, 32]. It is noteworthy that both Y191H and A437S mutations were reported in Dd2 strain which is highly resistant to CQ [51].

### 3.2. *Pf*crt (*Plasmodium falciparum* Chloroquine Resistance Transporter) as an Essential Tool in Conferring Chloroquine Resistance

Later on, another protein was discovered on the surface of the DV membrane called* pf*crt (*Plasmodium falciparum* chloroquine resistance transporter). It is a 48 KDa putative transporter or channel that belongs to DMT family of transporter proteins (drug metabolite transporter). It acts as an anion channel and mediates CQ efflux outside the DV. This role that has been studied extensively in lots of* in vitro* cross-genetic transfection studies ended up with controversial results. Some of them proved the dependency of CQR on* pf*crt expression level while others failed [17, 52]. This discrepancy was first attributed to the inconsistency of the experimental conditions and the used strains [40]. Later on, this debate was solved as it was found that CQR is more related to point mutations in* pf*crt gene rather than* pf*crt expression level [53].

#### 3.2.1. *Pf*crt Structure

**Figure 2:**
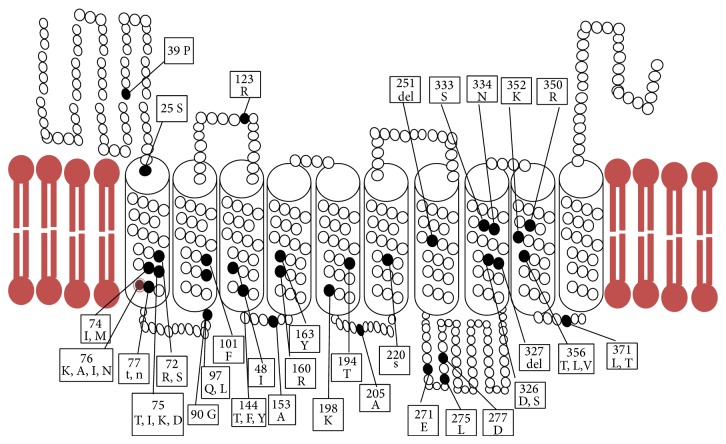
Detailed structure of* pf*crt protein. It is made up of 422 amino acids distributed over 10 transmembrane domains. Inside the structure there are 32 candidate codons for having point mutations that confer for changing* pf*crt function. The majority of them occur at the site that faces the DV media. Binding of substrates to* pf*crt does not require ATP activation as in P-glycoprotein molecules.

*Pf*crt is made up of 424 amino acids arranged in 10 *α*-helical transmembrane domains (TDMs) oriented inside the DV membrane and N-termini which are exposed to the cytosol. TDMs contain active sites that mediate binding and translocation of substrates.  Figure 2 shows the detailed structure of* pf*crt. There is an internal symmetry between the first and second fives TDMs such that TDMs 4 and 9 are responsible for binding and translocation of substrates. Both 3 and 8 assist in binding and translocation of the substrates and affect substrate specificity. TDMs 1, 2, 6, and 7 are responsible for identifying the substrates while 5 and 10 are responsible of homodimer formation.

#### 3.2.2. *Pf*crt Function

It is suggested that* pf*crt performs several functions, such as efflux of alkaloids, amine compounds, divalent cations and amino acids, and peptides that result from the vacuolar digestion of globin. Furthermore, it is culminated to have a role in regulation of H^+^ homeostasis [53].

#### 3.2.3. *Pf*crt Mutations and CQR

Most of the studies that were concerned about investigating the relationship between* pf*crt and CQR are confounded by inaccuracies that stemmed from relying on one time dose response measurement and using polyclonal fresh clinical isolates [54].

Till now, 32 plausible point mutations have been identified in* pf*crt gene. Their ubiquity alters* Pf*crt physiochemical properties and the phenotype of the strain regarding CQ susceptibility [55].

#### 3.2.4. Effect of Amino Acids Substitution on* Pf*crt Physiochemical Properties

Any point mutation that alters amino acid sequence of any channel results in changing its physiochemical properties and functional characters. Each amino acid has certain distinguishing properties that determine its impact on the channel function, such as molar mass, van der Waals volume and Vre (average volume of buried residue), lipophilicity or hydrophobicity index, and isoelectric point [56]. These properties affect channel function through changing its side chain volume, negativity, and polarity. The first three amino acids properties are related to the tendency of the amino acid to create a bulky group that hinders passage of the entities through the channel. Van der Waals volume refers to an imaginary hard sphere that the molecule occupies. Meanwhile, *V*_*r*_^*e*^ is related to the tendency of the amino acid to be buried inside the protein and is calculated from the area of its side chain. On the other hand, lipophilicity index is an index that refers to the ability of the amino acids to associate with lipophilic molecules. Meanwhile, isoelectric point (IP) refers to the pH at which the amino acid forms the zwitterion [56] (Table 3).

Normally, DV pH is maintained at narrow range which is more or less around 5. The majority of the mutations take place at the side that faces the cytosol. Accordingly, those amino acids, whose IP is more than 5, tend to impart positivity of for the channel. When IP value approaches or drops below than 5, higher channel negativity is conferred. Consequently, this affects CQ exodus outside the channel [57].

#### 3.2.5. K76T Mutation

In K76T mutation, the neutral threonine replaces the positively charged lysine residue at codon 76 in the structure of* pf*crt [58, 59]. It is a marker of CQR as its ubiquity is provisional to confer CQ resistance [60]. In contrary, there are few exceptions wherein, in spite of presence of K76T, the parasite is CQ susceptible. This is due to coexistence of other unique mutations that obviate K76T effect [61]. As an exception, J9 strain, which is found in Thailand, has got K76A mutation in which alanine is present instead of threonine at codon 76 [18].

Lysine replacement by threonine at codon 76 imparts for higher lipophilicity and negativity in* pf*crt lining and reduces the side chain volume. These changes favor egress of the ionized fraction of CQ (CQH^+^ & CQH_2_^++^) outside the DV [62]. Due to its positive charge, lysine hampers egress of CQ through repulsing the positively charged ionized CQ molecule. Moreover, it has a bulkier side chain compared to threonine which hinders CQ egress. Furthermore, this change increases the size of the hydrophobic sites which are required by CQ to bind into the channel before it egresses out. Accordingly, the majority of the scientists favored the third hypothesis to interpret the role of K76T mutation in conferring CQR [60].

Discovery of 106/I clone of* Plasmodium falciparum *is a clear evidence of the importance of K76T mutation in conferring CQR as this strain contains 6 out of 7 well-known mutations that are consistently present in the CQ resistant cohorts except for K76T. Absence of K76T turned 106/I to a CQ susceptible parasite [62, 63].

K76T mutation is primarily and completely selected after long term exposure to CQ. On the other hand, its ubiquity is not correlated with LM, HF, ART, and MQ susceptibility. This discrepancy is due to the disparity of the mechanism through which resistance to each of these drugs develop [64].

#### 3.2.6. Other Corollary* Pf*crt Mutations

After discovering K76T mutation, other nonsilent point mutations were discovered in loci: 19, 58, 72, 74, 75, 90, 97, 101, 123, 140, 146, 148, 152, 160, 163, 194, 198, 205, 220, 251, 271, 275, 277, 326, 327, 333, 334, 356, 371, 350, and 352 (Tables 4 and 5) [54]. Rapid diagnostic assays for* pf*crt mutations are already employed as surveillance tools for drug resistance [65].

Various patterns of genetic mutations occur in different geographic areas due to the disparity in the condition that the parasite experiences. Majority of them occur at codons 72, 74, 75, and 76 (Table 4) [66]. These variations result in lots of structural polymorphic changes in* pf*crt with appearance of several haplotypes with different degree of CQ resistance, CQ resistance by VPL and other reversing agents [17], and susceptibility to other antimalaria drugs.

It was found that eleven out of the total 32 polymorphic residues are associated with CQR, such as M74I, N75E, K76T, H97Q, A220S, Q271E, N326S, I356T,C350S, and R371I R. At least another three mutations are required along with K76T to confer CQ resistance [18] (Tables 4 and 5).

Various* pf*crt mutations are found in different geographic loci. This discrepancy is attributed to the difference in the history of the drug use in different places which result in evolution of alternate sets of* pf*crt mutations [67, 68] (Tables 4 and 5).

Genetic studies, which had screened the CQR related genetic foci and chromosomal markers in different geographic areas, found that* Plasmodium falciparum *strains isolated from Southeast Asia and Africa (old world strains) have quite similar major resistance to foci. On the other hand, the strains in each of South America and Papua New Guinea (new world strains) strains arose independently and each one has its own drug resistance genetic foci [69, 70]. Tables 6 and 7 contain a list of the most famous CQ resistant and susceptible strains and their geographic distribution.

VPL induced CQ resistance reversibility is more pronounced in old world strains (Dd2 and K76I) while it is absent in South American ones, namely, 7G8. Scientists had attributed this to the ability of VPL to interact with* pf*crt of the former and its failure to do so with the latter [59].

The majority of the effective mutations (Tables 4, 6, and 7) occur in the region between codons 72 and 76. This had resulted in evolution of different genotypic sequences, namely, CVMNK, CVIET, and SVMNT, which consequently produce different phenotypes with different aptitude to confer CQR (Tables 4, 6, and 7). CVMNK is a characteristic feature of CQ susceptible parasites. Both CVIET and SVMNT are found in CQ resistant strains of* Plasmodium falciparum*. The former is distributed in Southeast Asia and the latter is found in Africa (Tables 6 and 7). Other mutations are found in loci distant from (72–76) region, namely, 97, 220, 271, 326, 356, and 371 (Tables 5, 6, and 7).

In the absence of K76T mutation, the other corollary mutations start affecting CQ response to a certain extent. Bayomi et al. found that CQ response in 106/I is less as compared to other CQ susceptible strains. The team attributed this to the presence of the other corollary mutations in* pf*crt that may impart for less CQ accumulation in the DV. Although 106/I harbors the mutant* pf*mdr-1, it does not impart for the reduced CQ response [63], such that, in one cross-genetic study, it was found that replacing the mutant* pf*mdr-1 allele in 106/I by pfmdr-1^D10^ allele, the wild* pf*mdr-1 subtype, or by any other wild* pf*mdr-1 allele did not produce any change in CQ susceptibility [71].

#### 3.2.7. Relationship between* Pf*crt and* Pf*mdr-1 Alleles

CQ resistant strains of* Plasmodium falciparum* possess codon mutations that alter* pf*crt structure along with microsatellite polymorphs that flank* pf*crt structure. CQ resistance mediated by the mutant* pf*crt is either modulated by another gene, which is* pf*mdr-1 gene that is linked to change of IC_50_ values of some strains, or by factors that create a physiological environment that prevent* pf*crt of exerting its full capacity of CQ resistance induction. Several cross-genetic and epidemiology studies run in Africa, Southeast Asia, and Oceania proved this impact. This relationship was proved in a cross-genetic study between clones from Brazil and Ghana, 7G8 and GB4 [72]. Response to CQ and AQ and to their respective metabolites, MDCQ (monodesthyl chloroquine) and MDAQ (monodesthyl amodiaquine), was measured with different* pf*mdr-1 and* pf*crt alleles. The study showed that* pf*crt allele is the major determinant in their response and the mutant alleles of* pf*mdr-1 can merely modulate the degree of resistance in the strains that express the mutant allele of* pf*crt. The study showed the importance of coexistence of* pf*mdr^7G8^ along with* pf*crt^7G8^ to confer higher degree of resistance to CQ, AQ, and their metabolites. Transfecting* pf*mdr-1^GB3^ allele to 7G8 strains reduces resistance to these drugs. The study determined contribution of each of 7G8* pf*mdr-1 mutations, 1034C, 1042D, and 1246Y in conferring CQR and AQR. It was found that 1042D has the major contribution in imparting moderate CQR, poor sensitivity to VPL, and high degree of AQR. In a study of CQR and AQR in PGN isolates, lots of CQR isolates were obtained carrying a* pf*crt with SVMNT genotype. Degree of their CQR is lower in the isolates that carry neither 86Y nor 1042D mutations in* pf*mdr-1 allele [72].

WHO graded resistance to early treatment failure, late treatment failure, or inadequacy of clinical and physiological response. Prevalence of treatment failure or drug resistance is concomitant with K76T and is augmented when* pf*mdr-1^N86Y^ allele coexists [73].

#### 3.2.8. Functional Role of* Pf*crt Mutation

Mutations in certain active foci of* pf*crt result in changing its aptitude to pump CQ and other drugs outside the DV and regulate the intravacuolar pH. Furthermore, it affects the ability of verapamil (VP) or other drugs to reverse CQ resistance [69].

*Pf*crt acts as an ion channel that regulates the electrochemical potential cross the DV membrane and regulates egress of the ionized drugs [74]. On the other hand,* pf*crt is related to V type ATPase enzyme. An enzyme is present in various organelle membranes and is involved in pumping of protons into the organelles.

#### 3.2.9. Regulation of DV pH and Effect of* Pf*crt Mutation on Intravacuolar pH

DV acidity is attributed to the ingress of H^+^ through V-type-ATPase [75] and H^+^-pyrophosphatase mediated H^+^ pump mechanisms [76]. DV alkalinization is attained by inhibiting of the former by concanamycin A or bafilomycin A1 and the latter by NaF [76]. H^+^ accompanies CQ while the latter is pumped outside the DV. The intravacuolar localization of chloroquine gives rise to a substantial leakage of H^+^ outside the DV in CQ resistant unsusceptible strains [77].

PH dependency of CQ-heme interaction confers for the importance of alkalinization to reverse CQR. If the reversal agent fails to alkalinize DV and promote exodus of H^+^, then reversal of the resistance fails to proceed.

Presence of the mutant* pf*crt in CQ resistance strains of* Plasmodium falciparum *is accompanied by a decrease in the intravacuolar pH [77, 78]. In one study, after transfecting a* pf*crt gene into an oocyst of* Xenopus laevis*, there was a significant rise in the intracytosolic and reduction of DV pH values due to role of* pf*crt in pumping H^+^ outside the DV [77, 78]. Meanwhile, other studies found that* pf*crt acts as a Cl^−^ channel and this augments its role in maintaining intravacuolar pH [70].

#### 3.2.10. *Pf*crt Mutations and Verapamil (VPL) Reversibility of CQ Resistance (Verapamil Effect (VE))

CQR reversal by verapamil (VPL) became another hallmark of CQR in the resistant strains of* Plasmodium falciparum*. Abundance of the hydrophobic sites in* pf*crt structure increases the chance of VPL-induced CQR reversal. VPL binds to the hydrophobic sites and acts as a bulky group preventing egress of CQ outside the cell. The bound verapamil can replace the lost lysine during the mutation and acts as a repulsing moiety for CQ [79].

Scientists started to investigate the correlation between any of the abovementioned channel physiochemical characters (see Section 3.2.3) and parasite susceptibility to CQ and DCQ (desethylchloroquine) and VE. The results were quite controversial as some could not find any correlation and they attributed the phenomena to factors related to spatial orientation of the active sites within the channel. Others found a correlation between side chain volume of the channel lining and susceptibility to CQ or DCQ and between hydrophobicity of the channel and VPL induced CQR reversal. This suggests that CQR requires presence of bulky groups within the channel that act as obstacles preventing CQ exodus outside the DV. On the other hand, VP induced reversal of CQR required presence of hydrophobic sites where VP bind and prevent exodus of CQ [80].

## 4. Chloroquine (CQ) Resistance as a Continuous Trait with Multifactorial Inheritance

Ubiquity of the mutant form of* pf*crt confers for higher CQR with an extent depending on the presence of other genetic loci [80]. It was found in a cross-genetic study between 3D7 (CQ susceptible strain in Southeast Asia) and 7G8, a South American CQ resistant strain of* Plasmodium falciparum*, that transfection of the mutant* pf*crt into the CQ susceptible parasite confers for higher CQ resistance in that parasite. However, the extent of resistance in the transfected parasite did not match with the CQ resistant strain. Nevertheless, this phenomenon does not imply to all CQ susceptible strains as performing the same transfection on D10 strains did not produce any change in CQ IC_50_ of the transfected parasite. On the other hand, expression of* pf*crt^7G8^ in both 3D7 and D10 had conferred for higher resistance to MDCQ (monodesethyl chloroquine). Interestingly, both CQ and MDQ (monodesethyl chloroquine) resistances after the transfection were VPL reversible. Nevertheless, VPL reversibility is absent in the Latin American strains of* Plasmodium falciparum*. This suggests that introduction of* pf*crt^7G8^ allele to other CQ sensitive strains may produce a VPL irreversible phenotype of CQ resistance. This suggests that VPL, as a calcium channel blocker, induces intracellular physiological changes in the VPL sensitive strains resulting in change of* pf*crt function and thence VPL reversibility of CQR. This point requires further investigation to find the precise effect of VPL in both Latin American and Southeast Asian strains of* Plasmodium falciparum *[81].

Mutant* pf*crt allele introduction does not merely change CQ IC_50_; it changes the slop of dose response curve with an evidence of continued growth at higher concentration. This effect is particularly pronounced after introducing* pf*crt^7G8^ allele to D10 (D10^pfcrt7G8^). This change had generated a phenotype characterized by high CQ tolerance which is indicated by CQ IC_99_ or IC_99_ values. Recrudescence is another mode of treatment failure as it was found that 50% of the* in vitro* cultures recrudesce after 6 days of CQ discontinuation. Introduction of* pf*crt^7G8^ allele to 3D7, D10, and GCO3 has raised level of recrudescence after CQ discontinuation. This suggests that the mutant allele of* pf*crt confers for tolerance as well as for resistance.

Fitness cost is another phenomenon related to long term discontinuation of the antimalaria drug. It was found that some resistant strains of* Plasmodium falciparum* revert to CQ susceptible ones after removal of the selection pressure. For instance, in Malawi, Dd2 strains lost their* pf*crt^dD2^ after drug discontinuation.

Detection of G224 and H209 in French Guinea isolates provides indisputable evidence that K76T is insufficient alone to confer CQR. Both G224 and H209 have got* pf*crt and* pf*mdr haplotypes identical to that of 7G8 with only difference of* pf*mdr-1 at codon 1034 of* pf*mdr and codon presence of C350R mutation in H209. Western blot analyses did not detect any difference in the copy number of* pf*crt allele. CQ activity screening study showed that these changes turned both G224 and H209 to CQ sensitive parasites as their IC_50_ values were comparable to that of CQ sensitive strains and less than that of 7G8. On the other hand, analysis of CQ dose response curve revealed a skew in CQ IC_90_ for both G224 and H209 toward that of 7G8. Furthermore, unlike other CQ sensitive strains, both G224 and H209 showed a comparable degree of recrudescence to that of 7G8. It was found that charge substitution at position 350 in* pf*crt was selected by QN pressure in CQ resistant cell line. It was accompanied by reversion of the cell line into CQ susceptible one. H209 shows higher level of resistance to QN and ART as compared to G224 and 7G8.

## 5. Effect of* Pf*crt and* Pf*mdr-1 on Other Antimalaria Drugs 

### 5.1. Effect on Amodiaquine (AQ)

Previous screening and cross-genetic* in vitro* studies showed that unlike CQ, amodiaquine (AQ) efficacy was not affected by the presence of any mutant form of* pf*crt. First this had suggested that certain structural features present only in CQ are required for drugs to bind to* pf*crt. Nevertheless, scientists found that AQ action* in vivo* is poorly correlated with its action* in vitro*.* In vivo*, AQ is metabolized to desethylamodiaquine (DAQ), an active metabolite to which the discrepancy in AQ action is attributed [80].

Unlike AQ, DAQ action is affected by ubiquity of the mutant form of* pf*crt. Presence of K76T mutation in* pf*crt facilitates escape of DAQ outside the digestive vacuole or its access to the 72–76 region of* pf*crt structure. This is due to loss of the lysine residue that adds the due positivity which is required to prevent exodus of positively charged 4-aminoquinoline moieties. Moreover, DAQ exodus was correlated to presence of VE or presence of higher hydrophobicity in the 72–76 regions of* pf*crt.* Pf*crt channel hydrophobicity especially at the regions 72–76 and VE is highly prominent in the Asian Old World strains of* Plasmodium falciparum* in comparison to the New World South American ones. Leaked DAQ molecules tend to bind to such hydrophobic sites creating a bottleneck that prevent exodus of further molecules. This characteristic makes DAQ susceptibility highly correlated with hydrophobicity of* pf*crt channel in the resistant strains of* Plasmodium falciparum*. Consequently, DAQ resistance is seen in the New World resistant strains of* Plasmodium falciparum* and is absent in the Old World ones [80].

### 5.2. Effect on Amantadine Action

Amantadine AM, an antiviral drug used for treatment and prophylaxis of influenza A virus infection, showed an anti-Plasmodium effect which is quite variable among different strains of* Plasmodium falciparum*. The action was more pronounced against the Asian strains Dd2 and J3D4, intermediate against the American strain 7G8, and nearly negligible in CQ sensitive strains [61, 82].

It was found that AM blocks M2 ion channels which is one of the viral envelope proteins that affects viral replication through regulation of H^+^ ingress into the virion after its endocytosis by the host cell. Point mutations that result in AM resistance was detected in M2 channels of influenza A virus. This suggests that AM may have an effect on the electrochemical gradient and proton pump of DV membrane [83, 84]. The studies revealed that* pf*crt and its K76T mutation do not merely have a determinant role on the differential pattern of CQ susceptibility but they have as much important role on that of amantadine as well [61, 84].

In Singh Sidhu et al.'s 2002 study, the endogenous* pf*crt allele of GCO3 was replaced by various* pf*crt^CQR^ alleles, such as those of Dd2, J3D4(K76I) and 7G8, to get recombinant clones that contain all genetic materials of GCO3 along with one of the* pf*crt^CQR^ alleles. The clones were different from the parent GCO3 strain as their degree of CQR was comparable to that of the original resistant cell lines that bear the transfected* pf*crt^CQR^ alleled [53]. Aftermath, several studies found that as these clones develop higher resistance to CQ, they develop higher susceptibility to other drugs as MQ, HF, and AM as well. This confirms that the degree of AM susceptibility is highly dictated by* pf*crt mutation. One of the plausible annotations of this is that expression* pf*crt^CQR^ on the surface of DV results in higher exodus of alkaline drugs outside the vacuole and this raises their intracytosolic concentration and lowers their concentration in DV. It is noteworthy to note that all of the abovementioned drugs are weak alkaline. Their nonionized fraction permeates into the vacuole by simple diffusion and once they reach the acidic milieu of DV, they ionize and trap there.* pf*crt^CQR^ alleles excrete them outside the vacuole resulting in either more intensified action if the drug target is in the cytosol or weaker action if it is in DV. MQ, HF, and AM targets are located in the cytosol so* pf*crt^CQR^ intensifies their action while CQ target is located in the DV so* pf*crt^CQR^ confers for its resistance [85, 86].

Along with three-month intermittent exposure of K1H6/2 strain, which harbors the same Dd2* pf*crt^CQR^ allele, to AM, AM resistance had been developed in a stepwise manner along with parallel improvement in CQ susceptibility [3] and without any effect on both HF and MQ susceptibility. Aftermath, this phenotype was dubbed as K1AM and the genetic sequence and expression level of its* pf*crt and* pf*mdr alleles were studied extensively. It was revealed that the mutational changes in K1AM were not related to* pf*mdr as neither mutational change nor change in expression level had been detected [87]. Interestingly, in spite of presence of both K76T and A220S mutations, K1AM was CQ susceptible. It is considered as the first validated example of CQ susceptible* Plasmodium falciparum *cell line expresses both of these mutations. DNA sequencing studies revealed two novel mutations in the* pf*crt allele of K1AM strain, S163R and I356V. The former is unique to K1AM as it has never been reported in any* Plasmodium falciparum *but the latter was detected in some strains and was culminated as a culprit for development of CQR [87, 88].

Bray et al. 2000 studied the effect of AM on CQR in K1H6/2 strain. They found that along with its anti-Plasmodium effect against CQ resistant cell lines of* Plasmodium falciparum,* AM has a dichotomous relationship with CQ. From one side, it acts as VPL as it reversed VPL reversible CQR in K1H6/2 strains and from the other side its intermittent use on K1H6/2 induces mutational changes that invert CQR and convert the strain into K1AM strain [89].

### 5.3. Relationship of CQR and Mefloquine Action

It was noted that CQ action contravenes that of MQ in most strains of* Plasmodium falciparum*. MQ susceptibility is higher in CQ resistant strains and vice versa. Scientists attributed this either to the discrepancy in the location of their target sites as MQ target is located in the cytosol while CQ's is localized in the DV or to the plausible direct effect of both of* pf*crt or* pf*mdr on MQ. Direct action of* pf*crt on MQ was excluded as it was found that MQ does not affect efflux of H^+^ from* pf*crt. Unlike CQ resistance, MQ resistance is more affected by* pf*mdr-1 rather than* pf*crt mutation [90]. Moreover, it is correlated with amplification of the wild form of* pf*mdr-1 that mediates MQ accumulation inside the DV. On the other hand, MQ susceptibility is either associated with* pf*mdr-1 deamplification or overexpression of* pf*mdr-1^CQR^. Furthermore, MQ resistance contravenes CQR that it is conferred by mutant form of* pf*mdr while* pf*crt just modifies it [90, 91].

S163R mutation rose independently in resistance selection experiments using HF or AM on K1H6/2 strains. It was detected in one CQ susceptible isolate that is dubbed aftermath as* pf*163 during a screening study for its ubiquity in 44 different geographic areas. Aftermath, through DNA sequencing studies, genetic sequence of* pf*crt allele of* pf*163 was compared with that of other plasmodium strains. It was found that all of its codons were quite similar to those of K1H6/2 strain except codon 163 which exhibits S163R mutation. This augments the notion that S163R mutation obviates CQ resistance in CQ resistant strains of* Plasmodium falciparum *[87, 88].

MQ resistance aroused prominently in the Cambodian-Thai and Thai-Mynamar borders during the last 10 years. Genotype mapping in these areas failed to find any correlation between MQ resistance and prevalence of K76T polymorph of* pf*crt. This suggests that MQR develops in a mechanism distant from that of CQ resistance [92].

### 5.4. Effect on Phenanthrenes

Halofantrine HF and lumefantrine (LM) are the two substituted phenanthrene classes of antimalaria compounds. Their precise mechanism of action has not been identified yet but it is suggested that they bind to hematin and to plasmepsin (hemoglobin degrading enzyme) [93].

 K1HF strain is an experimental strain obtained by exerting a selection pressure of HF on K1H/6/2 strain.* In vitro* intermittent exposure of K1 cell line to HF results in higher resistance to MQ and HF and loss of CQR. This strain does not show any mutational change in* pf*mdr allele while a prominent change is seen in* pf*crt.* Pf*crt keeps both K76T and A220 S mutations but it gains other unique ones, such as T152A, S163R, and P275L. The scientists attribute most of the changes in CQ susceptibility to S163R as it was expressed in K1AM and both of them possess the same phenotypic pattern regarding CQ susceptibility in two independent experiments [94].

LM is mostly given in combination with artemether (ART) (AL-treatment) under a trade name Coartam. Coartam has been used extensively in Africa due to high spread of CQ resistance. According to a study in Kenya, which had investigated the relationship between the* in vitro* susceptibility of different antimalarials with different* pf*crt and* pf*mdr polymorphs, susceptibility of* Plasmodium falciparum* to LM is inversely proportional to that of CQ with a significant correlation. Furthermore, expression of wild type of* pf*crt and* pf*mdr, which are known for imparting higher CQ susceptibility, suppresses LM susceptibility [95].

It was found that long term exposure to LM may select for more CQ susceptible and LM resistant strains as it selects for the wild type of* pf*mdr-1 and increase its copy number. Emergence of LM resistance is more pronounced in the strains harboring the wild form of* pf*crt along with the wild form of* pf*mdr-1 [87]. It is noteworthy to dictate that such genotype is highly susceptible to CQ and this suggests the inverse relationship between CQ and LM. The rate of LM resistance emergence may occur rapidly. Unlike CQ, previous cross-genetic and transfection studies revealed that expression of the mutant* pf*crt in* Plasmodium falciparum* has less impact on the geometric mean of LM IC_50_ as compared to that of the mutant* pf*mdr. Similarly, both LM and MQ are unlike CQ in that they are rather more affected by* pf*mdr-1 than by* pf*crt mutation. Consequently, LM requires the mutant form of* pf*mdr-1 to enhance its access into the DV where it is thought to produce its action [95, 96].

### 5.5. Effect on Dihydroartemisinin (DHA)

It was found that DHA susceptibility was higher for strains harboring the wild rather than the mutant genotype of* pf*mdr-1. Meanwhile, its susceptibility was enhanced in the presence of the mutant genotype of* pf*crt. This urged for DHA use to treat CQ resistant strains of* Plasmodium falciparum *[95]. According to a cross-genetic study run by Andriantsoanirina et al. 2009, in which transfecting of different CQ sensitive strains with* pf*crt^7G8^ allele enhanced DHA susceptibility [97]. This mutation is not stable as it can be affected by drugs selection pressure. For instance, evolution of H209 from 7G8 strain which possessed C350R mutation due to the selection pressure of QN. The mutation reverted the strain into ART resistant strain as well.

### 5.6. Effect on Quinine Sensitivity

Both quinine (QN) and quinidine (QD) are famous antimalaria compounds that belong to quinoline family. It is thought that their target site of action is inside the DV. Their sensitivity is highly related to K76T mutation of* pf*crt. For instance, 106/I strains, a CQ sensitive strain present in Sudan and is characterized by absence of* pf*crt^K76T^, are insensitive to both QN and QD [62].

Long term selection pressure of CQ on 106/I had induced either K76I or K76N mutation in* pf*crt. Ubiquity of K76I turned the parasite into CQ resistant and produced a unique stereospecific response as it was turned into highly QN sensitive (17-folds decrease in IC_50_) and QD insensitive (2-folds increase in IC_50_) parasites. Interestingly, VPL reduced QN sensitivity and raised QD sensitivity in the mutated parasite. 106/I can expel QN outside the DV but this aptitude is lost once K76I is introduced. On the other hand, introduction of K76I mutation into 106/I had increased its* pf*crt potency to expel QD extravacuolarly. This augments the direct interaction of these drugs with* pf*crt [87].

Griffin et al. had investigated the impact of* pf*crt mutations on antimalaria effect of cinchona alkaloids (C.A). C.A contain quinoline amino alcohols exemplified by quinine, hydroquinone, cinchonine, epiquinine, quinidine, hydroquinidine, cinchonidine, and epiquinidine [98]. Each of them contains two chiral centers located at positions 8 and 9. The first four species belong to erythroisomers while the next four are dextrorotatory isomers. Both dextrorotatory isomers were more potent toward CQ sensitive strains rather than the erythroisomers. Consequently, it was concluded that* pf*crt is the major determinant of this stereospecific action. Furthermore, lysine ubiquity at position 76 in the wild form of* pf*crt may prevent exodus of only dextrorotatory isomers through steric repulsion. The ionic group in the dextrorotatory isomers can face the 76 lysine in the wild form of* pf*crt resulting in creation of a steric hindrance that hinders their exodus. On the other hand, erythroisomers dodge this steric hindrance as they face the 76 lysine residue of* pf*crt through their hydrophobic part. As an exception, epiquinine and epiquinidine failed to show any effect against* Plasmodium falciparum* as they lose the aptitude to inhibit *β* hematin formation. Exodus of cinchona alkaloids is inversely proportional to the positivity of* pf*crt channel. On the other hand, anti-Plasmodium effect of the erythroisomers was quite similar in both CQ sensitive and resistant strains of* Plasmodium falciparum* but the difference was for the dextrorotatory ones. This supports the determinant role of* pf*crt in the stereospecific action of C.A. The possibility that* pf*mdr mutations affect this stereospecificity was excluded as there was no any correlation between these stereoisomers anti-Plasmodium activity and ubiquity of different* pf*mdr-1 mutation. Furthermore, there was no effect of stereoisomerism on binding of any of the cinchona quinoline alkaloids with *β* hematin [98].

### 5.7. Effect on Amodiaquine (AQ)

Amodiaquine is a 4-aminoquinoline antimalaria drug whose mechanism of action is similar to CQ. It is effective against the Asian and African CQ resistant strains of* Plasmodium falciparum* but is ineffective against the South American ones. The latter are moderate CQ resistant and highly resistant to AQ. It was recommended by WHO to be used as a part of artemisinin combination therapy (ACT) in Africa [99]. The precise effect of both* pf*crt and and* pf*mdr on AQ resistance is still unclear. It was found that the* pf*crt allele of the African strains is more linked to AQ resistance rather than* pf*mdr-1 as cross-genetic studies revealed that the degree of resistance was similar when different* pf*mdr-1 alleles had been transfected as partners with the African* pf*crt^CQR^ allele [35]. 

### 5.8. Effect on Piperaquine

Previously, it was dictated that PIP IC_50_ is high in CQ resistant strains of* Plasmodium falciparum*. In a cross-genetic study, where* pf*crt^7G8^ allele was introduced into different CQ susceptible strains, it was found that it confers for higher PIP resistance. As an exception, PIP sensitivity was high in G224, a CQ sensitive strain carrying the same* pf*crt^7G8^, and D10^*pf*crt7G8^, a CQ susceptible strain transfected with* pf*crt^7G8^ allele and H209, which carries a mutant* pf*crt^7G8^ allele with C350R mutation [54].

## 6. Conclusion 

It is noteworthy to point out that the problem of drug resistance to antimalarials is quite horrendous due to continuous emergence of drug resistant strains. Both* pf*mdr-1 and* pf*crt determine susceptibility of* Plasmodium falciparum* to antimalarials as they control the amount of the drug that accumulates inside the digestive vacuole. Efficacy of the drugs whose target site of action is intravacuolar increases if the abovementioned transporters shuffle them into the digestive vacuole and vice versa for those whose target site is extravacuolar. There is a close positive association between responses of* Plasmodium falciparum* to MQ, HF, LM, and DHA. These responses are inversely proportional to those of CQ. These changes are conferred by ubiquity of both* pf*crt and* pf*mdr-1 mutations. This observation suggests the presence of a common element of multigenic mechanism.

Studying the correlation between drug resistance in the parasites and genetic polymorphism may allow for developing new tools to predict responses to drugs.

## Figures and Tables

**Table 1:** List of plausible mutations in *pf*mdr-1 of *Plasmodium falciparum * along with properties of both substituent and substituted amino acids.

Site of mutation	Substituted amino acids	Polarity	Side chain charge	Hydrophobicity index	Substituent amino acid	Side chain polarity	Side chain charge	Hydrophobicity index
86	Asparagine	Polar	Neutral	−3.5	Tyrosine	Polar	Neutral	−1.3
1042	Asparagine	Polar	Neutral	−3.5	Aspartic acid	Acidic polar	Negative	−3.5
1034	Serine	Polar	Neutral	−0.8	Cystien	Non polar	Neutral	2.5
1246	Aspartic acid	Acidic polar	Negative	−3.5	Tyrosine	Polar	Neutral	−1.3

**Table 2:** List of mutations in *pf*mrp of *Plasmodium falciparum* along with properties of both substituent and substituted amino acids.

Site of mutation	Substituted amino acids	Side chain polarity	Side chain charge	Hydrophobicity index	Substituent amino acid	Side chain polarity	Side chain charge	Hydrophobicity index
191	Tyrosine	Polar	Neutral	−1.3	Histidine	Basic polar	Partially positive	−3.2
437	Alanine	Non polar	Neutral	1.8	Serine	Polar	Neutral	−0.8

**Table 3:** Physiochemical properties of different amino acids.

Amino acid	Code	Formula	Molar mass	Van der Waals volume	*V*_*r*_^*e*^ *A* ^3^	Polarity	Acidity	Hydropathy index	Isoelectric point (pI)
Alanine	Ala/A	C_3_H_7_NO_2_	89.09	67	92	Nonpolar	Neutral	1.8	6.01
Arginine	Arg/R	C_6_H_14_N_4_O_2_	174.2	148	225	Polar	Basic (strong)	−4.5	10.76
Asparagine	Asn/N	C_4_H_8_N_2_O_3_	132.11	96	135	Polar	Neutral	−3.5	5.41
Aspartic acid	Asp/D	C_4_H_7_NO_4_	133.1	91	125	Polar	Acidic	−3.5	2.85
Cysteine	Cys/C	C_3_H_7_NO_2_S	121.15	86	106	Polar	Neutral	2.5	5.05
Glutamic acid	Glu/E	C_5_H_9_NO_4_	147.13	109	161	Polar	Acidic	−3.5	3.15
Glutamine	Gln/Q	C_5_H_10_N_2_O_3_	146.15	114	155	Polar	Neutral	−3.5	5.65
Glycine	Gly/G	C_2_H_5_NO_2_	75.06	48	66	Nonpolar	Neutral	−0.4	6.06
Histidine	His/H	C_6_H_9_N_3_O_2_	155.15	118	167	Polar	Basic (weak)	−3.2	7.6
Isoleucine	Ile/I	C_6_H_13_NO_2_	131.17	124	169	Nonpolar	Neutral	4.5	6.05
Leucine	Leu/L	C_6_H_13_NO_2_	131.17	124	168	Nonpolar	Neutral	3.8	6.01
Lysine	Lys/K	C_6_H_14_N_2_O_2_	146.18	135	171	Polar	Basic	−3.9	9.6
Methionine	Met/M	C_5_H_11_NO_2_S	149.2	124	171	Nonpolar	Neutral	1.9	5.74
Phenylalanine	Phe/F	C_9_H_11_NO_2_	165.19	135	203	Nonpolar	Neutral	2.8	5.49
Proline	Pro/P	C_5_H_9_NO_2_	115.13	90	129	Nonpolar	Neutral	−1.6	6.3
Serine	Ser/S	C_3_H_7_NO_3_	105.09	73	99	Polar	Neutral	−0.8	5.68
Threonine	Thr/T	C_4_H_9_NO_3_	119.12	93	122	Polar	Neutral	−0.7	5.6
Tryptophan	Trp/W	C_11_H_12_N_2_O_2_	204.22	163	240	Nonpolar	Neutral	−0.9	5.89
Tyrosine	Tyr/Y	C_9_H_11_NO_3_	181.19	141	203	Polar	Neutral	−1.3	5.64
Valine	Val/V	C_5_H_11_NO_2_	117.14	105	142	Nonpolar	Neutral	4.2	6

**Table 4:** List of the plausible mutations that may occur in *pf*crt within the regions 72–76 along with their geographic distribution, effect on channel physiochemical properties, and their impact on the phenotype of the parasite toward chloroquine resistance.

Mutation	Geographic distribution	Effect on channel negativity	Effect on side chain volume	Effect on the channel lipophilicity	Overall effect on chloroquine resistance and VPL induced reversal of CQ resistance
C72S	CQ resistant strains of the new world	High increase		Decrease	Imparts for non VPL reversible CQ resistance

N74I	Old World CQ resistant strains	High increase		Increase	Imparts for VPL reversible CQ resistance

N75D	Cambodian CQ resistant strains	Increase	Decrease		Imparts for higher CQR without affecting VPL binding

N75E	Old World CQ resistant strains	Increase		No effect	Imparts for higher CQR without affecting VPL binding

K76N	Long term exposure of 106/I strain to CQ	Increase			Imparts for VPL reversible CQR with IC_50_ of about 12-folds that of 106/I. On the other hand, it imparts for higher MQ, HF, LM, and DHA activity

K76I	Long term exposure of 106/I strain to CQ	Increase			Imparts for higher CQR with IC_50_ of about 12-folds that of 106/I. On the other hand, it imparts for higher MQ, HF, LM, and DHA activity

**Table 5:** List of some famous mutations in *pf*crt in foci far from 72 to 76 regions along with their geographic distribution, effect on channel physiochemical properties, and their impact on the phenotype of the parasite toward chloroquine resistance.

Mutation	Geographic distribution	Effect on channel negativity	Effect on side chain volume	Effect on the channel lipophilicity	Overall effect on chloroquine resistance and VPL induced reversal of CQ resistance
H97Q	Only in TM90-6CB, the Thai CQ resistant strain	Increase		No effect	Imparts for higher CQ resistance without affecting VPL binding

A220S	All CQ resistant strains	Increase		Decrease	Impart for non-VPL reversible CQ resistance

Q271E	Old World CQ resistant strains and the CQ susceptible strains derived from KH1 strain	Increase			Imparts for higher CQ resistance without affecting VPL binding

N326S	Old World CQ resistant strains and the CQ susceptible strains derived from KH1 strain			Increase	Imparts for reversibility of CQ resistance by VPL

I356T	Some CQ resistant strains in Southeast Asia, namely, Dd2, BC7, BC22, KS28, and 738	Increase	Decrease	Decrease	Impart for non-VPL reversible CQ resistance (it explains why VPL reversibility is higher in K1 rather than in Dd2)

I356L	New World CQ resistant strains	Little increase		Little increase	Imparts for higher CQ resistance and VPL induced reversibility of CQ resistance

R371	Old World CQ resistant strains and the CQ susceptible strains derived from KH1 strain			Increase	Impart for VPL induced reversibility of CQ resistance

**Table 6:** List of chloroquine sensitive strains of *Plasmodium falciparum* along with their geographic distribution and both *pf*crt and *pf*mdr-1 haplotypes.

Strain	Geographic distribution	*Pf*crt haplotype																							*Pf*mdr-1				
		*72 *	*74 *	*75 *	*76 *	*97 *	*123 *	*144 *	*148 *	*152 *	*160 *	*163 *	*194 *	*198 *	*205 *	*220 *	*271 *	*275 *	*277 *	*326 *	*333 *	*350 *	*356 *	*371 *	*86 *	*184 *	*1034 *	*1042 *	*1246 *
***D7*** (***wild type***)	Southeast Asia and Africa	C	M	N	K	H	H	A	L	T	L	S	I	E	T	A	Q	P	N	N	T	C	I	R	N	Y	S	N	D
***HB3***	Honduras	C	M	N	K	H	H	A	L	T	L	S	I	E	T	A	Q	P	N	N	T	C	I	R	N	Y	S	D	D
***GC03***	Genetic-cross studies of Dd_2_ and HB_3_	C	M	N	K	H	H	A	L	T	L	S	I	E	T	A	Q	P	N	N	T	C	I	R	N	Y	S	N	D
***MP2475***	Malaysia	C	M	N	K	H	H	A	L		L		I	E	T	A			N	N	T		I		N	Y	S	N	D
***MP2533***	Malaysia	C	M	N	K	H	H	A	L		L		I	E	T	A			N	N	T		I		N	F	S	N	D
***N29.07***	Africa (Nigeria)	C	M	N	K	H	H	A	L		L		I	E	T	A			N	N	T		I		N	F	S	N	D
***N60 and N92***	Africa (Nigeria)	C	M	N	K	H	H	A	L		L		I	E	T	A			N	N	T		I		N	Y	S	N	D
Th230.08	Africa (Senegal)	C	M	N	K	H	H	A	L		L		I	E	T	A			N	N	T		I		N	Y	S	N	D
Tg060.07	Africa (Senegal)	C	M	N	K	H	H	A	L		L		I	E	T	A			N	N	T		I		N	F	S	N	D
***P09.04 and P19.04***	Africa (Pikine/Senegal)	C	M	N	K	H	H	A	L		L		I	E	T	A			N	N	T		I		N	Y	S	N	D
***P31.01 and P08.04***	Africa (Pikine/Senegal)	C	M	N	K	H	H	A	L		L		I	E	T	A			N	N	T		I		N	F	S	N	D
***P11.02***	Africa (Pikine/Senegal)	C	M	N	K	H	H	A	L		L		I	E	T	A			N	S	T		I		N	F	S	N	D
***P27.02***	Africa (Pikine/Senegal)	C	M	N	K	H	H	A	L		L		I	E	T	A			N	N	T		I		Y	F	S	N	D
***P60.02***	Africa (Pikine/Senegal)	C	M	N	K	H	H	A	L		L		I	E	T	S			N	N	T		I		N	F	S	N	D
***K1 AM***	Positive selection of K1H with amantadine	C	I	E	T	H	H	A	L	T	L	R	I	E	T	S	E	P	N	S	T	C	V	I					
***K1 HF***	Positive selection of K1H with HF	C	I	E	T	H	H	A	L	A	L	R	I	E	T	S	E	L	N	S	T	C	I	I					
***Pf164***	Southeast Asia	C	I	E	T	H	H	A	L	T	L	R	I	E	T	S	E	P	N	S	T	C	I	I					
***106/I***	Sudan (Awad-el-Kariem FM 1992)	C	L	E	K	H	H	A	L	T	L	S	I	E	T	S	E	P	N	S	T	C	I	I					
***D10***	Papau New Guinea	C	M	N	K	H	H	A	L	T	L	S	I	E	T	A	Q	P	N	N	T	C	I	R	N	Y	S	N	D
***D6***	Sierra Leone	C	M	N	K	H	H	A	L	T	L	S	I	E	T	A	Q	P	N	N	T	C	I	R	Y	Y	S	N	D
***NF54***		C	M	N	K		H	A	L		L		I	E	T				N		T				N	Y	S	N	D
***H209***	French Guinea	S	M	N	T	H	H	A	L	T	L	S	I	E	T	S	Q	P	N	D	T	R	L	R					

**Table 7:** List of chloroquine resistant strains of *Plasmodium falciparum* along with their geographic distribution and both *pf*crt and *pf*mdr-1 haplotypes.

Strain	Geographic distribution	CQR reversal by VPL	*Pf*crt haplotype																							*Pf*mdr-1				
			*72 *	*74 *	*75 *	*76 *	*97 *	*123 *	*144 *	*148 *	*152 *	*160 *	*163 *	*194 *	*198 *	*205 *	*220 *	*271 *	*275 *	*277 *	*326 *	*333 *	*350 *	*356 *	*371 *	*86 *	*184 *	*1034 *	*1042 *	*1246 *
***CQ resistant strains***								H	A	L		L		I	E	T				N		T								
***K1H***	Southeast Asia (Thailand)	✓	C	I	E	T	H	H	A	L	T	L	S	I	E	T	S	E	P	N	S	T	C	I	I					
***J9***	Southeast Asia (Thailand)	✓	C	I	E	A	H	H	A	L	T	L	S	I	E	T	S	E	P	D	S	T	C	I	I					
***BC7***	Thailand		C	I	E	T	H	H	A	L	T	L	S	I	E	T	S	E	P	N	S	T	C	T	I					
***BC22***	Thailand		C	I	E	T	H	H	A	L	T	L	S	I	K	T	S	E	P	N	S	T	C	T	I					
***KS28***	Thailand		C	I	E	T	H	H	A	L	T	L	S	I	E	T	S	E	P	N	S	T	C	T	I					
***Dd2***	Southeast Asia (Indochina and Laos)	✓	C	I	E	T	H	H	A	L	T	L	S	I	E	T	S	E	P	N	S	T	C	T	I					
***K76I*** (***J34***)	Southeast Asia	✓	C	I	E	I	H	H	A	L	T	L	S	I	E	T	S	E	P	N	S	T	C	I	I					
***PH1***	The Philippines		C	M	N	T	H	H	T	L	T	Y	S	I	E	T	A	Q	P	N	D	T	C	I	R					
***PH2***	The Philippines		S	M	N	T	H	H	T	L	T	Y	S	I	E	T	A	Q	P	N	D	T	C	I	R					
***738***	Cambodia		C	I	D	T	H	H	A	I	T	L	S	T	E	T	S	E	P	N	N	T	S	I	R					
***734***	Cambodia		C	I	D	T	H	H	A	I	T	L	S	T	E	T	S	E	P	N	N	T	S	I	R					
***783***	Cambodia		C	I	E	T	H	H	A	L	T	L	S	I	E	T	S	E	P	N	D	T	S	T	I					
***TM90-6CB***	Thailand	✓	C	I	E	T	Q	H	A	L		L	S	I	E	T	S	N		N	S	T		I		N	F	C	D	Y
***TM6***	Thailand							**R**	A	L		L		I	E	K				N		T								
***TM93-C1088***	Thailand							H	A	L		L		I	E	T				N		T								
***2300***	INDONESIA		C	I	K	T	H	H	A	L	T	L	S	I	E	T	S	E	P	N	S	T	S	I	I					
***PNG4***	PNG		S	M	N	T	H	H	A	L	T	L	S	I	E	T	A	Q	P	N	D	T	C	L	R					
***APO41***	Africa (Nigeria)	✓	C	I	E	T	H	H	A	L		L		I	E	T	S			N	N	T		I		N	F	S	N	D
***N60***	Africa (Nigeria)	✓	C	I	E	T	H	H	A	L		L		I	E	T	S			N	N	T		I		N	Y	S	N	D
***N62***	Africa (Nigeria)	✓	C	I	E	T	H	H	A	L		L		I	E	T	S			N	N	T		I		Y	F	S	N	D
***P05.02 and P51.02***	Africa (Pikine/Senegal).	✓	C	I	E	T	H	H	A	L		L		I	E	T	S			N	S	T		I		Y	F	S	N	D
***P26.02***	Africa (Pikine/Senegal)	✓	C	I	E	T	H	H	A	L		L		I	E	T	S			N	N	T		S		Y	F	S	N	D
***GB4***	Ghana		C	I	E	T	H	H	A	L	T	L	S	I	E	T	S	E	P	N	N	T	S	I	R					
***7G8***	South America (Brazil)	X	S	M	N	T	H	H	A	L	T	L	S	I	E	T	S	Q	P	N	D	T	C	L	R	N	F	C	D	Y
***J3D4***								H	A	L		L		I	E	T				N		T								
***G209***	South America	X	S	M	N	T	H	H	A	L	T	L	S	I	E	T	S	Q	P	N	D	T	R	L	R	N	F	S	D	Y
***G224***	South America	X	S	M	N	T	H	H	A	L	T	L	S	I	E	T	S	Q	P	N	D	T	C	L	R	N	F	S	D	Y
***TU741***	Colombia		C	M	N	T	H	H	A	L	T	L	S	I	E	T	S	Q	P	N	D	T	N	L	R					
***JAV***	Colombia		C	M	E	T	H	H	A	L	T	L	S	I	E	T	S	Q	P	N	N	T	S	I	T					
***TA7519***	Colombia		C	M	E	T	Q	H	A	L	T	L	S	I	E	T	S	Q	P	N	N	T	S	I	T					
***TA6182***	Colombia		C	M	E	T	Q	H	A	L	T	L	S	I	E	T	S	Q	P	N	S	T	S	I	T					
***ECU1110***	Ecuador		C	M	N	T	H	H	A	L	T	L	S				S	Q	P	N	D	T	S	L	R					
